# Pluralistic care pathways may compound care delays and health inequalities across urban, rural, and pastoralist Somaliland

**DOI:** 10.3389/fpubh.2026.1961529

**Published:** 2026-09-24

**Authors:** Abdifatah Ahmed Osman, Hamze G. Dahir

**Affiliations:** 1 School of Public Health and Nutrition, College of Health Sciences, Amoud University, Borama, Somalia; 2 Research and Innovation Center, Amoud University, Borama, Somalia

**Keywords:** healthcare access, health-seeking behavior, medical pluralism, pastoralists, referral systems, social inequalities, Somaliland, universal health coverage

## Abstract

Health-seeking behavior, healthcare access, and health inequalities in Somaliland are shaped by medical pluralism: people may move among traditional or faith healers, pharmacies and drug sellers, private clinics, and hospitals during the same illness episode. These pluralistic care pathways may differ across urban, rural, and pastoralist communities, yet they are rarely measured as complete therapeutic itineraries. This Perspective develops a conceptual argument, grounded in published literature and policy documents, that the public health concern is not pluralism itself but poorly connected transitions that may accumulate time, cost, and clinical risk before appropriate care is reached. We introduce pluralistic delay as a testable construct and integrate Kleinman's medical sectors, Andersen's Behavioral Model, Levesque's patient-centered access framework, and the Three Delays model into a Somaliland Pluralistic Care Pathway and Delay Framework. The framework hypothesizes fragmented abundance in urban settings, constrained escalation in rural settings, and mobility-amplified delay among pastoralist populations; these patterns are propositions for empirical testing, not estimates of prevalence or causal effects. We propose an equity-oriented “regulate and connect” agenda: risk-based pharmacy regulation, red-flag referral partnerships with trusted community providers, mobile and corridor-based services for pastoralists, referral-linked transport and financing, and routine measurement of pre-facility care pathways. The approach may inform universal health coverage and is consistent with Sustainable Development Goals 3, 10, and 17, the WHO Global Traditional Medicine Strategy 2025–2034, Somaliland's national health and development plans, and African Union Agenda 2063.

## 1 Introduction

In many low-resource settings, the first response to illness is not a formal clinical encounter. People interpret symptoms within households and communities, try familiar remedies, consult religious or traditional practitioners, buy medicines directly, and seek biomedical care when illness persists, worsens, or is recognized as dangerous. This coexistence and use of multiple healing systems is commonly described as medical pluralism (1, 2). In the Somali cultural region, ethnographic work has documented prayer, traditional therapies, pharmaceuticals, and biomedical services within the same illness narratives rather than as mutually exclusive therapeutic worlds (2).

Somaliland is an important setting for examining the public health consequences of these pathways. Its National Health Policy III recognizes that a large share of the population seeks care from a largely unregulated private sector that includes pharmacies, clinics, hospitals, and traditional healers, while also identifying limited access for nomadic populations and weaknesses in regulation and referral (3). Yet access is commonly assessed at the final point of contact. A person recorded as a hospital user may have spent days moving through household treatment, a healer, and one or more medicine sellers before arrival. This obscures where preventable delay, financial exposure, and inequality accumulate.

Pluralism should not be equated with irrationality or harm. In Somaliland, repositioning traditional birth attendants (TBAs) as birth companions and promoters of skilled attendance showed that trusted traditional actors can become effective referral bridges when facilities are prepared to receive them (4). Recent qualitative work across Somaliland similarly describes TBAs as trusted intermediaries between rural communities and the formal health system (5). The relevant question is therefore not how to eliminate pluralism, but how to make plural pathways safer, earlier, and more connected.

Existing scholarship has described therapeutic itineraries and therapy management across plural medical systems, while health-systems research has separately examined continuity, fragmentation, navigation, medicine retail, and referral performance (6–12). This Perspective builds incrementally on those traditions. Its proposed contribution is to isolate the potentially avoidable time, cost, and clinical risk generated at transitions between unlike provider sectors; to treat settlement ecology and mobility as modifiers of those transition costs; and to specify indicators through which these propositions can be tested in urban, rural, and pastoralist Somaliland. It does not claim that pluralism invariably produces delay or that these constructs have already been validated.

## 2 Conceptualizing pluralistic care pathways and delay

Medical pluralism refers here to the coexistence and use of more than one healing tradition, provider type, or therapeutic logic within the same health environment or illness episode (1, 2). A therapeutic itinerary is the temporal sequence through which an individual or household interprets symptoms, chooses providers, evaluates response, switches treatment, and eventually recovers, disengages from care, or reaches a higher-level service. These concepts shift analysis from a single provider choice to a dynamic care pathway.

The neighboring concepts are related but not interchangeable. Therapeutic-itinerary research follows how patients and therapy-managing groups interpret illness, select among alternatives, and revise decisions over time (8). Continuity and coordination of care concern whether information and responsibility are connected across encounters, while fragmentation denotes failures in those connections and is especially consequential when patients move across levels or sectors (9, 10). Patient navigation is an organized support function intended to help people overcome barriers (11).

Research on medicine retailers and referral systems often focuses on a particular entry point or transfer (7, 12). Pluralistic delay adds a narrower temporal and equity proposition: avoidable delay may accumulate specifically at repeated cross-sector transitions. Pathway-adjusted access is the corresponding assessment lens for the cumulative burden of the whole itinerary. Their incremental value therefore lies in specifying the transition as the unit of analysis and in testing whether its consequences vary by settlement ecology and mobility, rather than in renaming medical pluralism or general access barriers.

These four established frameworks provide interlocking components for our model.

Kleinman's sectors map the spatial and cultural terrain (where care is sought) (1). Andersen's Behavioral Model identifies the individual and household drivers prompting movement between these sectors (why care is sought) (13). Levesque's framework defines the friction at each provider interface (how easily a transition occurs) (14). And the Three Delays model provides the temporal metric for the entire journey (how much time is lost). Together, they explain how a patient moves through plural sectors, driven by behavioral needs, constrained by access barriers at each node, and accumulating time deficits (15).

We integrate these frameworks to define pluralistic delay as a proposed, testable construct: the cumulative avoidable delay that may arise when a single illness episode is navigated sequentially or cyclically across providers without timely resolution or effective escalation. Conceptually, it separates delay associated with provider transitions—such as duplicated assessment, non-referral, failed referral, or treatment switching—from baseline delays caused by geography or transport, while recognizing that the two may interact. “Appropriate care” is defined non-hierarchically as care that is safe, effective, culturally acceptable, and clinically timely for a specific condition; depending on the illness, this may involve a biomedical facility or a recognized traditional practitioner working within an appropriate community and referral-governance arrangement. Evidence from eastern and southern Africa indicates that plural care may either facilitate engagement or create bottlenecks when referral and treatment are poorly coordinated (6).

Pathway-adjusted access is proposed as a complementary assessment lens rather than a validated scale. It extends conventional measures centered on the availability, distance, or price of the final facility by also examining the total time, cumulative cost, number and type of contacts, referral completion, and clinical transitions across the full therapeutic itinerary. Unlike therapeutic itinerary, which primarily describes the sequence and social logic of care seeking, pathway-adjusted access evaluates the burden and equity consequences of that sequence. Unlike patient navigation, which is usually an intervention to help patients overcome barriers, it is a measurement perspective for identifying where navigation fails (8, 9, 11, 12).

## 3 Global, African, Horn of Africa, and Somaliland context

Globally, traditional, complementary, and integrative medicine has moved toward a formal agenda of evidence, safety, regulation, and appropriate health-system integration. The WHO Global Traditional Medicine Strategy 2025–2034 calls for stronger evidence, enhanced safety and quality, appropriate integration into health systems and universal health coverage, and cross-sectoral partnerships (16). This agenda supports culturally respectful care while making clear that integration must be evidence-informed and safety-oriented.

Across sub-Saharan Africa, pluralistic pathways reflect both cultural legitimacy and structural constraints. Traditional and faith healers may be trusted and geographically accessible, while formal and informal medicine retailers frequently serve as first entry points to health systems (6, 7). Research on referral systems and continuity further shows that a referral is not a completed transfer: communication, transport, affordability, receiving-facility readiness, and feedback all influence whether care is actually continued (9, 12, 23–25). For nomadic pastoralists, systematic reviews suggest that conventional fixed-facility models often fit poorly with mobility, sparse infrastructure, and seasonal livelihoods (17, 18). Taken together, this literature supports the plausibility of the proposed framework, but it does not establish the prevalence or comparative magnitude of pluralistic delay in Somaliland.

The Horn of Africa adds a distinctive interaction between mobility, climate variability, conflict histories, humanitarian intervention, and pharmaceutical markets. In the Somali Region of Ethiopia, pharmaceuticals, prayer, and traditional practices coexist within therapeutic itineraries (2). In Somaliland, pastoral movement changes with environmental conditions and conflict, making geographic access dynamic rather than static (19).

Somaliland's policy architecture already recognizes many of the underlying problems. National Health Policy III emphasizes equitable access, referral, regulation, public-private coordination, and health-system research (3). National Development Plan III 2023–2027 prioritizes improved coverage of essential services, referral strengthening, better regulation of private health services, health information, and community engagement (20). A pathway perspective therefore builds on existing policy commitments rather than proposing a parallel system.

## 4 Social inequalities across urban, rural, and pastoralist pathways

We define “settlement ecology” not as a fixed geographic residence, but as a dynamic, overlapping state of livelihood, seasonal mobility, and sedentarization that can change during a single illness episode. The following patterns—“fragmented abundance,” “constrained escalation,” and “mobility-amplified delay”—are presented as analytical hypotheses requiring empirical validation rather than proven epidemiological patterns for Somaliland. Table 1 summarizes the resulting hypotheses.

**Table 1:** Proposed mechanisms of pluralistic care delay and inequality across Somaliland settlement ecologies.

Settlement ecology	Likely pathway feature	Dominant inequality/delay mechanism	Priority public health response
Urban	Dense mix of pharmacies, private clinics, laboratories, and hospitals; multiple entry points	Fragmented navigation, repeated payment or treatment, weak referral continuity, variable quality	Pharmacy regulation; red-flag triage; referral directories; connected public-private pathways
Rural	Fewer formal services; local traditional actors, TBAs, and medicine outlets may be more accessible	Serial escalation plus transport delay; late referral; affordability constraints	Community referral partnerships; rural transport; strengthened primary care; feedback to referrers
Pastoralist	Mobile livelihoods and long, seasonally changing distance to fixed facilities	Mobility-amplified delay: failed contacts or referrals multiply travel, opportunity, and financing costs	Mobile/outreach care; corridor-based service points; emergency transport and financing; portable referral information

These mechanisms are hypotheses derived from the cited literature and policy context; they are not prevalence estimates for Somaliland.

### 4.1 Urban Somaliland

Cities and larger towns generally have denser networks of pharmacies, private clinics, laboratories, and hospitals. Physical proximity may therefore coexist with navigational fragmentation. Where medicines can be purchased before diagnostic assessment, patients may move between retail treatment and clinical care, paying repeatedly while the underlying condition remains unrecognized. Evidence from sub-Saharan Africa shows the wider importance of medicine retailers as first-contact providers, including informal outlets (7). Urban pluralistic delay is therefore likely to arise from fragmented entry points, variable quality, weak referral continuity, and affordability rather than distance alone.

### 4.2 Rural Somaliland

Rural communities have fewer nearby services and greater transport constraints. Traditional practitioners, TBAs, small medicine outlets, and community networks may consequently be practical first contacts. A pathway can escalate from household care to a trusted local actor, then to a health center, pharmacy, or higher-level facility. Each step can be rational under local constraints yet still create cumulative delay. Somaliland's TBA experience demonstrates that the pathway can be redesigned… However, evidence concerning TBAs in obstetric care cannot be automatically generalized to infectious diseases, chronic conditions, or pediatric emergencies, which require distinct referral architectures (4, 5).

### 4.3 Pastoralist Somaliland

For mobile pastoralists, an additional transition may increase distance, transport, and opportunity costs, although the size and causal effect of this mechanism have not been established. Qualitative research among pastoral communities in Somalia describes long distances, transport constraints, and affordability barriers to maternal care (21). A recent mixed-methods study spanning Somalia and Somaliland also reported substantial access and financing difficulties across settlement groups (22). Neither study directly measured provider switching or validated between-group differences in pluralistic delay. They instead provide contextual evidence for the hypothesis that failed contacts or incomplete referrals may carry particular consequences for mobile households. We term this proposition mobilityamplified pluralistic delay.

## 5 Somaliland pluralistic care pathway and delay framework

Figure 1 translates the argument into a testable framework. Rather than a linear hierarchy ending at a hospital, the framework is conceptualized as a network. Illness begins in the popular sector, where symptoms are interpreted and initial action is taken. From there, individuals may remain in self-care, contact traditional or faith practitioners, obtain medicines from pharmacies or drug sellers, enter clinics or hospitals, or move bidirectionally and cyclically among these points. The pathway is modified by trust, explanatory models of illness, gender and household decision-making, health literacy, previous experience, price, provider density, opening hours, transport, seasonality, mobility, medicine availability, regulation, and perceived quality.

**Figure 1:**
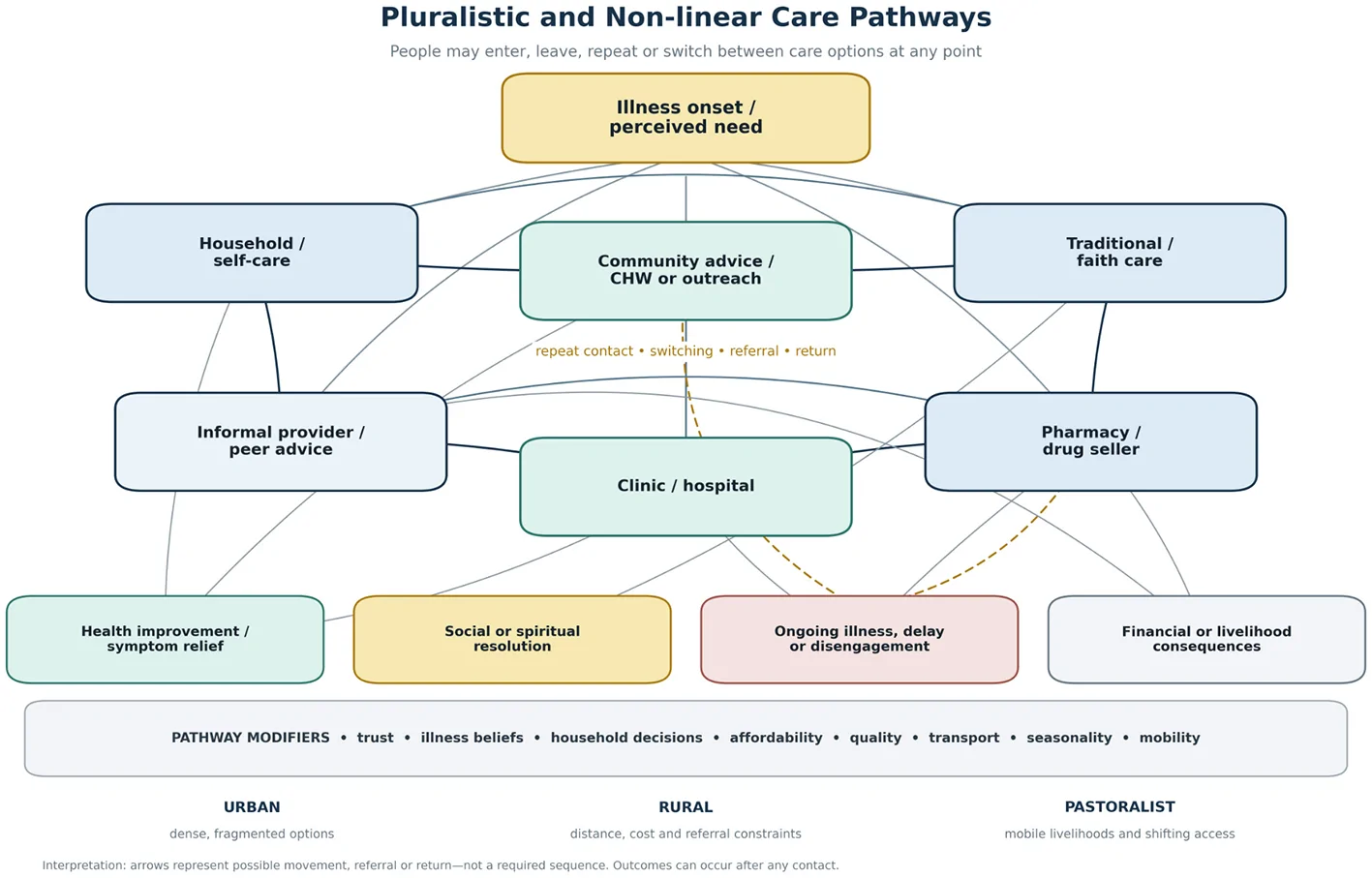
Somaliland Pluralistic Care Pathway and Delay Framework. The framework links household interpretation and self-care with traditional or faith providers, pharmacies or drug sellers, and biomedical facilities. Transitions can shorten delay when they trigger timely referral or lengthen delay when they create therapeutic cycling without appropriate escalation. Settlement ecology and social determinants modify the time, cost, and clinical consequences of each transition.

The primary outcome is time to appropriate care, defined condition-specifically. Secondary pathway measures should include the number and type of previous contacts, time spent at each stage, reasons for switching, direct and indirect costs, medicines received, whether a referral was issued and completed, and clinical severity on arrival. These measures distinguish a productive plural pathway—where an early actor accelerates referral—from therapeutic cycling in which repeated contacts postpone effective care.

The framework also shifts responsibility away from blaming patients for ‘late presentation.' Delay can be co-produced by system design. A household may seek help early but still reach appropriate care late because the first accessible provider lacks a referral protocol, a medicine seller has little incentive to redirect a customer, transport is unavailable, or the receiving facility is unaffordable. Pluralistic delay is therefore simultaneously behavioral, social, geographic, and governance-related.

## 6 Equity-oriented public health action: regulate and connect

A credible response should follow a regulate-and-connect principle. The aim is neither to endorse every traditional practice nor to medicalize all community care. It is to recognize the providers people already encounter, reduce avoidable risk, and create faster connections to appropriate care.

First, selected traditional and faith practitioners and TBAs could be linked to red-flag referral pathways. Crucially, this must not reduce traditional and faith leaders to mere unpaid auxiliaries of the biomedical system. Integration requires their active participation in protocol co-development, mutual recognition, bidirectional referral feedback, non-punitive relationships, and community governance that respects their cultural authority (4, 5).

Second, pharmacies and drug sellers should be treated as public health access points as well as commercial outlets. Priorities include licensing, minimum competency standards, inspection, antimicrobial stewardship, limits on unsafe dispensing, standardized danger-sign referral, and up-to-date referral directories. These measures are consistent with national commitments to regulate the private sector and improve referral (3, 20).

Third, referral design should be settlement-sensitive. Urban areas need connected triage and information; rural areas need dependable escalation routes and transport; pastoralist areas need mobile or outreach services, strategically located service points, and delivery schedules responsive to seasonal movement. Existing geospatial research shows that pastoral mobility in Somaliland can be modeled in relation to environmental and conflict conditions, creating opportunities for more adaptive planning (19).

Fourth, referral financing should be assessed from the beginning of the pathway, not only at the final facility. Transport vouchers, emergency referral funds, exemptions for priority conditions, community financing, or future risk-pooling mechanisms should be evaluated for their ability to reduce cumulative costs across multiple contacts.

Fifth, routine information systems should make the pre-facility pathway visible. A small dataset—first point of care, previous contacts, symptom-onset date, referral source, major medicines already taken, travel time, and settlement type—could identify conditions and population groups in which pathway delay and inequality are most pronounced.

## 7 Research priorities and future directions

The next step is empirical validation rather than further assumption. Prospective therapeutic-itinerary studies should enroll patients or households at symptom onset, or as close to onset as feasible, with purposive or stratified representation of urban, rural, and pastoralist communities. Conditions should include time-sensitive acute illness, maternal emergencies, common childhood illness, chronic disease, and mental health because the meaning and consequences of delay vary by condition.

A minimum dataset should record provider sequence, switching intervals, reasons for each choice, diagnostic and treatment actions, medicine exposure, referral completion, cumulative out-of-pocket and transport costs, time to appropriate care, and outcome. Mixed methods are essential: sequence and time data can quantify delay, while interviews can explain trust, explanatory models, gendered decision-making, perceived quality, and reasons for leaving or returning to providers. Analyses should test whether settlement ecology modifies the association between provider transitions and time to appropriate care.

The framework is also transferable beyond Somaliland. Similar pathway research could examine mobile populations, border communities, fragile settings, and places where medicine retailers and traditional practitioners function as important first contacts. Comparative work across the Horn of Africa could determine which mechanisms are context-specific and which represent broader forms of inequality in plural health systems.

## 8 Discussion

Somaliland's plural health system may be usefully conceptualized as a network through which people navigate illness rather than as a hierarchy in which traditional care, pharmacies, and hospitals occupy separate rungs. On the basis of the reviewed literature, pluralism could support resilience when accessible and trusted actors recognize limits and facilitate timely referral; conversely, repeated treatment, weak regulation, and poor referral may contribute to cumulative delay. The proposed concept of pluralistic delay makes this distinction explicit and links medical anthropology with behavioral theory, patient-centered access, continuity of care, and time-to-care models. These are conceptual propositions requiring prospective validation in Somaliland.

The urban–rural–pastoralist comparison is central to the proposed public health contribution, but it should be read as a set of hypotheses rather than demonstrated population differences. Urban residents may confront fragmented abundance, rural residents may experience constrained escalation, and pastoralists may face mobility-amplified access costs. If supported empirically, these would represent social and geographic inequalities in the pathway to care, not simply differences in facility density. The framework therefore suggests testing differentiated responses that regulate medicine retail, connect trusted community actors to formal services, finance completed referral, and bring appropriate services closer to mobile populations. By making pathway delay measurable, the framework could help operationalize Somaliland's National Development Plan III, the WHO Global Traditional Medicine Strategy, and SDG 3 while extending attention beyond facility-based access metrics.

The principal strength of this Perspective is its synthesis of established theory with Somali and Somaliland literature to generate explicit, measurable propositions rather than a purely descriptive account. It also treats settlement ecology as a potential effect modifier of access rather than a fixed background characteristic. Several limitations are important. This is a conceptual Perspective based on a targeted, non-systematic use of secondary literature and policy documents; it is not a systematic review, primary analysis, or causal study. The evidence base is heterogeneous across conditions and locations, and evidence from Somalia, the wider Horn of Africa, or sub-Saharan Africa may not transfer directly to Somaliland. Pluralistic delay and pathway-adjusted access have not been psychometrically or prospectively validated, and the manuscript does not estimate pathway prevalence, quantify associations between contact number and delay, establish urban–rural–pastoralist differences, or demonstrate a causal effect of mobility. The framework and settlement-specific patterns should therefore be treated as hypotheses for empirical testing.

## 9 Conclusion

Public health surveillance and policy should consider not only where people finally seek care but also the pathway taken before that contact. The proposed measures—number and type of contacts, time and cost at each step, referral issuance and completion, and clinical status on arrival—require prospective validation in Somaliland. If the hypothesized patterns are supported, pathway measurement could make avoidable delay more visible, inform safer and better-connected referral, and contribute to more equitable progress toward universal health coverage.

## Data Availability

The original contributions presented in the study are included in the article/supplementary material, further inquiries can be directed to the corresponding author.
